# 1-(1-Phenyl­ethyl­idene)carbonohydrazide

**DOI:** 10.1107/S1600536810038353

**Published:** 2010-09-30

**Authors:** Yan Qiao, Xiuping Ju, Zhiqing Gao, Lingqian Kong

**Affiliations:** a Dongchang College, Liaocheng University, Liaocheng 250059, People’s Republic of China

## Abstract

The title compound, C_9_H_12_N_4_O, crystallizes with two independent mol­ecules in the asymmetric unit. In the crystal, inter­molecular N—H⋯O and N—H⋯N hydrogen bonds link the mol­ecules into paired ribbons propagated in [100]. The crystal studied was a twin (twin law 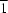
00/0
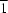
0/001) with a minor component of 25%.

## Related literature

For applications of carbonohydrazide derivatives, see: Esmail & Kurzer (1977 ▶); Loncle *et al.* (2004 ▶). For a related structure, see: Meyers *et al.* (1995 ▶).
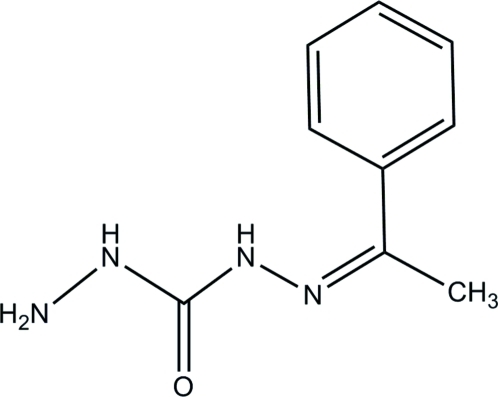


## Experimental

### 

#### Crystal data

C_9_H_12_N_4_O
*M* *_r_* = 192.23
Monoclinic, 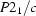

*a* = 9.7744 (8) Å
*b* = 7.3163 (7) Å
*c* = 28.2761 (3) Å
β = 90.796 (1)°
*V* = 2021.9 (3) Å^3^
*Z* = 8
Mo *K*α radiation
μ = 0.09 mm^−1^
*T* = 293 K
0.43 × 0.17 × 0.15 mm

#### Data collection

Bruker SMART APEX CCD area-detector diffractometer
Absorption correction: multi-scan (*SADABS*; Sheldrick, 2007 ▶) *T*_min_ = 0.963, *T*_max_ = 0.987
9568 measured reflections
3568 independent reflections
1412 reflections with *I* > 2σ(*I*)
*R*_int_ = 0.111

#### Refinement

*R*[*F* ^2^ > 2σ(*F* ^2^)] = 0.062
*wR*(*F* ^2^) = 0.186
*S* = 0.84
3568 reflections
256 parameters
H-atom parameters constrained
Δρ_max_ = 0.26 e Å^−3^
Δρ_min_ = −0.24 e Å^−3^

### 

Data collection: *SMART* (Bruker, 2007 ▶); cell refinement: *SAINT* (Bruker, 2007 ▶); data reduction: *SAINT*; program(s) used to solve structure: *SHELXS97* (Sheldrick, 2008 ▶); program(s) used to refine structure: *SHELXL97* (Sheldrick, 2008 ▶); molecular graphics: *SHELXTL* (Sheldrick, 2008 ▶); software used to prepare material for publication: *SHELXTL*.

## Supplementary Material

Crystal structure: contains datablocks I, global. DOI: 10.1107/S1600536810038353/cv2761sup1.cif

Structure factors: contains datablocks I. DOI: 10.1107/S1600536810038353/cv2761Isup2.hkl

Additional supplementary materials:  crystallographic information; 3D view; checkCIF report

## Figures and Tables

**Table 1:** Hydrogen-bond geometry (Å, °)

*D*—H⋯*A*	*D*—H	H⋯*A*	*D*⋯*A*	*D*—H⋯*A*
N2—H2⋯N8^i^	0.86	2.19	2.982 (4)	152
N4—H4*C*⋯O2^ii^	0.89	2.29	3.055 (5)	144
N3—H3⋯O2	0.86	2.13	2.895 (4)	148
N6—H6*A*⋯N4	0.86	2.17	2.972 (4)	156

Symmetry codes: (i) 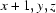; (ii) 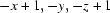.

**Fig. 1.**
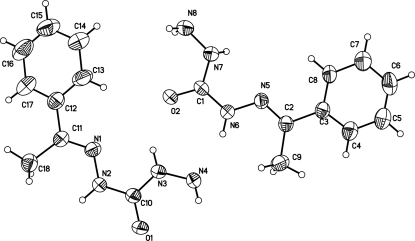
A view of (I) showing the atomic numbering scheme and 30% probability displacement ellipsoids.

## supplementary crystallographic information

### Comment

Carbonohydrazide derivatives are popular ligands in coordination chemistry due to the strong coordinative ability (Esmail *et al.*, 1977). Meanwhile, they have also attracted much attention due to interesting bioactivity such as antibacteriale antifungal, anticonvulsant, anticancer activities (Loncle *et al.*, 2004). Herewith we present the crystal structure of the title compound, (I), which is a carbonohydrazide derivative.

In (I) (Fig. 1), the bond lengths and angles are normal and comparable to those observed in the reported compound (Meyers *et al.*, 1995). The C=N bond lengths in the molecule are 1.282 (5) °, 1.272 (5)° (C2=N5, C1=N11), respectively, showing the double-bond character. The dihedral angle between the benzene ring (C12—C17) and the plane of C11/N1/N2  is 19.17 (27) °, while the dihedral angle between the benzene ring (C3—C8) and the plane of C2/N5/N6  is 14.87 (31) °.

Intermolecular N—H···O and N—H···N hydrogen bonds (Table 1) link the molecules into paired ribbons propagated in direction [100].

### Experimental

Acetophenone (10.0 mmol) and carbohydrazide (10.0 mmol) were mixed in 50 ml flash under sovlent-free condtions. After stirring 2 h at 373 K, the resulting mixture was cooled to room temperature, and recrystalized from ethanol, and afforded the title compound as a crystalline solid. Elemental analysis: calculated for C_9_H_12_N_4_O: C 56.24, H 6.29, N 29.15%; found: C 56.13, H 6.24, N 29.31%.

### Refinement

All H atoms were placed in geometrically idealized positions (N—H = 0.86–0.90 Å and C—H = 0.93 - 0.96 Å) and treated as riding on their parent atoms, with *U*_iso_(H) = 1.2–1.5 *U*_eq_(C, N).

### Crystal data

**Table:**

C_9_H_12_N_4_O	*F*(000) = 816
*M**_r_* = 192.23	*D*_x_ = 1.263 Mg m^−^^3^
Monoclinic, *P*2_1_/*c*	Mo *K*α radiation, λ = 0.71073 Å
*a* = 9.7744 (8) Å	Cell parameters from 839 reflections
*b* = 7.3163 (7) Å	θ = 2.5–26.2°
*c* = 28.2761 (3) Å	µ = 0.09 mm^−^^1^
β = 90.796 (1)°	*T* = 293 K
*V* = 2021.9 (3) Å^3^	Block, colourless
*Z* = 8	0.43 × 0.17 × 0.15 mm

### Data collection

**Table:**

Bruker SMART APEX CCD area-detector diffractometer	3568 independent reflections
Radiation source: fine-focus sealed tube	1412 reflections with *I* > 2σ(*I*)
graphite	*R*_int_ = 0.111
phi and ω scans	θ_max_ = 25.0°, θ_min_ = 1.4°
Absorption correction: multi-scan (*SADABS*; Sheldrick, 2007)	*h* = −10→11
*T*_min_ = 0.963, *T*_max_ = 0.987	*k* = −8→8
9568 measured reflections	*l* = −33→23

### Refinement

**Table:**

Refinement on *F*^2^	Primary atom site location: structure-invariant direct methods
Least-squares matrix: full	Secondary atom site location: difference Fourier map
*R*[*F*^2^ > 2σ(*F*^2^)] = 0.062	Hydrogen site location: inferred from neighbouring sites
*wR*(*F*^2^) = 0.186	H-atom parameters constrained
*S* = 0.84	*w* = 1/[σ^2^(*F*_o_^2^) + (0.0777*P*)^2^] where *P* = (*F*_o_^2^ + 2*F*_c_^2^)/3
3568 reflections	(Δ/σ)_max_ < 0.001
256 parameters	Δρ_max_ = 0.26 e Å^−^^3^
0 restraints	Δρ_min_ = −0.24 e Å^−^^3^

### Special details

**Table:**

Geometry. All e.s.d.'s (except the e.s.d. in the dihedral angle between two l.s. planes) are estimated using the full covariance matrix. The cell e.s.d.'s are taken into account individually in the estimation of e.s.d.'s in distances, angles and torsion angles; correlations between e.s.d.'s in cell parameters are only used when they are defined by crystal symmetry. An approximate (isotropic) treatment of cell e.s.d.'s is used for estimating e.s.d.'s involving l.s. planes.
Refinement. Refinement of *F*^2^ against ALL reflections. The weighted *R*-factor *wR* and goodness of fit *S* are based on *F*^2^, conventional *R*-factors *R* are based on *F*, with *F* set to zero for negative *F*^2^. The threshold expression of *F*^2^ > σ(*F*^2^) is used only for calculating *R*-factors(gt) *etc*. and is not relevant to the choice of reflections for refinement. *R*-factors based on *F*^2^ are statistically about twice as large as those based on *F*, and *R*- factors based on ALL data will be even larger.

### Fractional atomic coordinates and isotropic or equivalent isotropic displacement parameters (Å^2^)

**Table:**

	*x*	*y*	*z*	*U*_iso_*/*U*_eq_	
O1	0.8472 (3)	0.1813 (5)	0.45497 (9)	0.0877 (10)	
O2	0.3489 (2)	0.2384 (4)	0.50884 (9)	0.0731 (9)	
N1	0.6814 (3)	0.3386 (5)	0.55616 (12)	0.0627 (9)	
N2	0.7850 (3)	0.3042 (5)	0.52501 (12)	0.0655 (10)	
H2	0.8681	0.3330	0.5320	0.079*	
N3	0.6226 (3)	0.1926 (5)	0.47282 (11)	0.0746 (11)	
H3	0.5607	0.2305	0.4918	0.089*	
N4	0.5831 (3)	0.0984 (6)	0.43144 (11)	0.0793 (11)	
H4A	0.6347	0.1350	0.4075	0.119*	
H4C	0.5948	−0.0210	0.4360	0.119*	
N5	0.1757 (3)	0.0963 (5)	0.40633 (11)	0.0578 (9)	
N6	0.2815 (3)	0.1455 (5)	0.43539 (11)	0.0610 (9)	
H6A	0.3642	0.1413	0.4254	0.073*	
N7	0.1225 (3)	0.2228 (5)	0.49114 (11)	0.0762 (11)	
H7A	0.0802	0.1160	0.4849	0.091*	
N8	0.0871 (3)	0.2698 (6)	0.53820 (12)	0.0931 (13)	
H8A	0.1197	0.1869	0.5584	0.140*	
H8C	0.1239	0.3782	0.5448	0.140*	
C1	0.2557 (4)	0.2014 (6)	0.48027 (15)	0.0612 (11)	
C2	0.2023 (4)	0.0482 (5)	0.36361 (14)	0.0559 (10)	
C3	0.0833 (4)	−0.0052 (6)	0.33345 (14)	0.0583 (11)	
C4	0.0951 (5)	−0.0984 (6)	0.29212 (15)	0.0735 (12)	
H4	0.1824	−0.1264	0.2817	0.088*	
C5	−0.0154 (6)	−0.1537 (7)	0.26486 (17)	0.0882 (15)	
H5	−0.0020	−0.2153	0.2365	0.106*	
C6	−0.1446 (6)	−0.1168 (8)	0.27995 (18)	0.0965 (17)	
H6	−0.2203	−0.1574	0.2626	0.116*	
C7	−0.1616 (5)	−0.0192 (8)	0.32097 (18)	0.1011 (17)	
H7	−0.2494	0.0110	0.3306	0.121*	
C8	−0.0505 (5)	0.0345 (7)	0.34802 (15)	0.0789 (14)	
H8	−0.0639	0.0976	0.3761	0.095*	
C9	0.3434 (4)	0.0465 (6)	0.34318 (14)	0.0776 (13)	
H9A	0.3935	0.1513	0.3543	0.116*	
H9B	0.3368	0.0498	0.3093	0.116*	
H9C	0.3903	−0.0629	0.3529	0.116*	
C10	0.7539 (4)	0.2242 (6)	0.48324 (16)	0.0670 (12)	
C11	0.7111 (4)	0.4054 (6)	0.59662 (15)	0.0609 (11)	
C12	0.5943 (5)	0.4406 (6)	0.62839 (16)	0.0696 (12)	
C13	0.4626 (5)	0.4533 (7)	0.60913 (17)	0.0916 (16)	
H13	0.4496	0.4393	0.5767	0.110*	
C14	0.3502 (6)	0.4868 (8)	0.6376 (2)	0.119 (2)	
H14	0.2631	0.4944	0.6241	0.143*	
C15	0.3676 (7)	0.5084 (9)	0.6855 (2)	0.126 (2)	
H15	0.2926	0.5326	0.7045	0.151*	
C16	0.4968 (8)	0.4940 (8)	0.7055 (2)	0.116 (2)	
H16	0.5093	0.5049	0.7381	0.139*	
C17	0.6091 (6)	0.4628 (6)	0.67616 (17)	0.0891 (15)	
H17	0.6963	0.4571	0.6897	0.107*	
C18	0.8554 (4)	0.4505 (6)	0.61330 (15)	0.0754 (13)	
H18A	0.9043	0.3393	0.6198	0.113*	
H18B	0.8517	0.5230	0.6416	0.113*	
H18C	0.9015	0.5178	0.5891	0.113*	

### Atomic displacement parameters (Å^2^)

**Table:**

	*U*^11^	*U*^22^	*U*^33^	*U*^12^	*U*^13^	*U*^23^
O1	0.0453 (16)	0.145 (3)	0.073 (2)	0.0036 (17)	0.0070 (15)	−0.0160 (19)
O2	0.0450 (15)	0.106 (2)	0.0682 (19)	0.0043 (15)	−0.0040 (14)	−0.0133 (17)
N1	0.062 (2)	0.070 (3)	0.057 (2)	0.0059 (18)	0.0080 (18)	−0.0016 (19)
N2	0.0499 (19)	0.088 (3)	0.058 (2)	−0.0044 (18)	0.0016 (17)	−0.012 (2)
N3	0.044 (2)	0.114 (3)	0.066 (2)	0.0049 (19)	0.0020 (16)	−0.025 (2)
N4	0.055 (2)	0.115 (3)	0.068 (2)	0.002 (2)	−0.0024 (17)	−0.015 (2)
N5	0.055 (2)	0.062 (2)	0.056 (2)	0.0020 (17)	−0.0064 (17)	−0.0019 (18)
N6	0.0501 (19)	0.080 (3)	0.054 (2)	−0.0011 (17)	0.0018 (16)	−0.0103 (19)
N7	0.054 (2)	0.111 (3)	0.064 (2)	−0.011 (2)	0.0074 (17)	−0.020 (2)
N8	0.056 (2)	0.152 (4)	0.072 (3)	0.002 (2)	0.0065 (18)	−0.015 (3)
C1	0.041 (2)	0.079 (3)	0.064 (3)	−0.003 (2)	−0.003 (2)	−0.002 (2)
C2	0.061 (3)	0.049 (3)	0.057 (3)	0.000 (2)	−0.004 (2)	0.001 (2)
C3	0.071 (3)	0.054 (3)	0.050 (2)	0.002 (2)	−0.003 (2)	0.006 (2)
C4	0.087 (3)	0.066 (3)	0.068 (3)	0.004 (3)	−0.005 (3)	−0.006 (3)
C5	0.116 (4)	0.078 (4)	0.069 (3)	−0.006 (3)	−0.016 (3)	−0.010 (3)
C6	0.105 (4)	0.104 (5)	0.079 (4)	−0.031 (4)	−0.027 (3)	0.003 (3)
C7	0.078 (4)	0.146 (5)	0.079 (4)	−0.013 (3)	−0.011 (3)	−0.004 (4)
C8	0.071 (3)	0.104 (4)	0.062 (3)	−0.011 (3)	−0.009 (2)	−0.009 (3)
C9	0.074 (3)	0.092 (4)	0.067 (3)	−0.001 (3)	0.011 (2)	−0.009 (3)
C10	0.050 (3)	0.089 (3)	0.062 (3)	0.009 (2)	−0.001 (2)	−0.006 (3)
C11	0.071 (3)	0.055 (3)	0.057 (3)	0.005 (2)	0.000 (2)	−0.001 (2)
C12	0.079 (3)	0.063 (3)	0.067 (3)	0.002 (2)	0.006 (2)	−0.002 (2)
C13	0.086 (4)	0.102 (4)	0.088 (4)	0.006 (3)	0.029 (3)	−0.021 (3)
C14	0.098 (4)	0.153 (6)	0.106 (4)	0.001 (4)	0.031 (4)	−0.032 (4)
C15	0.114 (5)	0.156 (6)	0.109 (5)	−0.011 (4)	0.052 (4)	−0.028 (4)
C16	0.153 (6)	0.122 (5)	0.074 (4)	−0.010 (5)	0.034 (4)	−0.016 (3)
C17	0.119 (4)	0.083 (4)	0.067 (3)	0.009 (3)	0.012 (3)	−0.005 (3)
C18	0.078 (3)	0.067 (3)	0.080 (3)	0.007 (2)	−0.009 (2)	−0.005 (2)

### Geometric parameters (Å, °)

**Table:**

O1—C10	1.260 (4)	C5—C6	1.365 (6)
O2—C1	1.239 (4)	C5—H5	0.9300
N1—C11	1.274 (4)	C6—C7	1.374 (7)
N1—N2	1.374 (4)	C6—H6	0.9300
N2—C10	1.349 (5)	C7—C8	1.377 (6)
N2—H2	0.8600	C7—H7	0.9300
N3—C10	1.333 (5)	C8—H8	0.9300
N3—N4	1.407 (4)	C9—H9A	0.9600
N3—H3	0.8600	C9—H9B	0.9600
N4—H4A	0.8900	C9—H9C	0.9600
N4—H4C	0.8900	C11—C12	1.485 (5)
N5—C2	1.288 (4)	C11—C18	1.518 (5)
N5—N6	1.361 (4)	C12—C17	1.366 (5)
N6—C1	1.360 (5)	C12—C13	1.393 (6)
N6—H6A	0.8600	C13—C14	1.393 (6)
N7—C1	1.351 (4)	C13—H13	0.9300
N7—N8	1.422 (4)	C14—C15	1.373 (7)
N7—H7A	0.9000	C14—H14	0.9300
N8—H8A	0.8900	C15—C16	1.380 (8)
N8—H8C	0.8900	C15—H15	0.9300
C2—C3	1.485 (5)	C16—C17	1.404 (7)
C2—C9	1.503 (5)	C16—H16	0.9300
C3—C4	1.359 (5)	C17—H17	0.9300
C3—C8	1.407 (5)	C18—H18A	0.9600
C4—C5	1.378 (6)	C18—H18B	0.9600
C4—H4	0.9300	C18—H18C	0.9600
			
C11—N1—N2	119.1 (3)	C8—C7—H7	119.6
C10—N2—N1	118.8 (3)	C7—C8—C3	120.5 (4)
C10—N2—H2	120.6	C7—C8—H8	119.8
N1—N2—H2	120.6	C3—C8—H8	119.8
C10—N3—N4	121.4 (3)	C2—C9—H9A	109.5
C10—N3—H3	119.3	C2—C9—H9B	109.5
N4—N3—H3	119.3	H9A—C9—H9B	109.5
N3—N4—H4A	109.4	C2—C9—H9C	109.5
N3—N4—H4C	109.1	H9A—C9—H9C	109.5
H4A—N4—H4C	109.5	H9B—C9—H9C	109.5
C2—N5—N6	118.5 (3)	O1—C10—N3	121.3 (4)
C1—N6—N5	119.6 (3)	O1—C10—N2	120.5 (4)
C1—N6—H6A	120.2	N3—C10—N2	118.2 (4)
N5—N6—H6A	120.2	N1—C11—C12	116.3 (4)
C1—N7—N8	119.3 (3)	N1—C11—C18	124.2 (4)
C1—N7—H7A	107.2	C12—C11—C18	119.6 (4)
N8—N7—H7A	106.0	C17—C12—C13	117.6 (4)
N7—N8—H8A	110.3	C17—C12—C11	123.1 (4)
N7—N8—H8C	107.9	C13—C12—C11	119.3 (4)
H8A—N8—H8C	109.5	C14—C13—C12	121.2 (5)
O2—C1—N7	121.9 (4)	C14—C13—H13	119.4
O2—C1—N6	122.0 (3)	C12—C13—H13	119.4
N7—C1—N6	116.0 (3)	C15—C14—C13	120.2 (6)
N5—C2—C3	116.4 (4)	C15—C14—H14	119.9
N5—C2—C9	124.1 (3)	C13—C14—H14	119.9
C3—C2—C9	119.5 (4)	C14—C15—C16	119.7 (5)
C4—C3—C8	116.5 (4)	C14—C15—H15	120.2
C4—C3—C2	123.4 (4)	C16—C15—H15	120.2
C8—C3—C2	120.1 (4)	C15—C16—C17	119.3 (5)
C3—C4—C5	123.6 (5)	C15—C16—H16	120.4
C3—C4—H4	118.2	C17—C16—H16	120.4
C5—C4—H4	118.2	C12—C17—C16	122.0 (5)
C6—C5—C4	119.2 (5)	C12—C17—H17	119.0
C6—C5—H5	120.4	C16—C17—H17	119.0
C4—C5—H5	120.4	C11—C18—H18A	109.5
C5—C6—C7	119.3 (5)	C11—C18—H18B	109.5
C5—C6—H6	120.3	H18A—C18—H18B	109.5
C7—C6—H6	120.3	C11—C18—H18C	109.5
C6—C7—C8	120.9 (5)	H18A—C18—H18C	109.5
C6—C7—H7	119.6	H18B—C18—H18C	109.5

### Hydrogen-bond geometry (Å, °)

**Table:**

*D*—H···*A*	*D*—H	H···*A*	*D*···*A*	*D*—H···*A*
N2—H2···N8^i^	0.86	2.19	2.982 (4)	152
N4—H4C···O2^ii^	0.89	2.29	3.055 (5)	144
N3—H3···O2	0.86	2.13	2.895 (4)	148
N6—H6A···N4	0.86	2.17	2.972 (4)	156

Symmetry codes: (i) *x*+1, *y*, *z*; (ii) −*x*+1, −*y*, −*z*+1.
